# Prevalence of Dental and Skeletal Fluorosis Among School Children in Rural Areas of YSR Kadapa District, Andhra Pradesh, India

**DOI:** 10.7759/cureus.51288

**Published:** 2023-12-29

**Authors:** Lavanya Sirigala, Pratibha Ramani, Priyadharshini G, Karthikeyan Ramalingam

**Affiliations:** 1 Oral Pathology and Microbiology, Saveetha Dental College and Hospitals, Saveetha Institute of Medical and Technical Sciences, Saveetha University, Chennai, IND

**Keywords:** ysr kadapa, skeletal system, teeth, children, drinking water, fluoride

## Abstract

Background

Andhra Pradesh (AP) is one of the states in India found to have districts with endemic fluoride and YSR Kadapa district is one among them. Fluorosis is a well-known endemic disease affecting the hard tissues of the human body like teeth and bone and soft tissues like the kidneys, gastrointestinal system, nervous system, etc. Dental fluorosis is being proven as a biomarker for exposure to fluoride. This study evaluated the toxicity of fluoride in drinking water on the teeth (dental fluorosis) and on the bones (skeletal fluorosis) in YSR Kadapa district, AP.

Material and methods

A cross-sectional study was done in schools in the study areas and a total of 488 school children aged between eight to 14 years were screened for fluorosis. Consent from parents or legal representatives was taken. The information regarding personal details, source of drinking water, diet, and socio-demographic characteristics was obtained from children through a questionnaire. Oral examination of teeth was done to assess dental fluorosis by the Deans's fluorosis index and a general physical examination to assess skeletal fluorosis as per guidelines given by the Central Government of India in the National Program for Prevention of Fluorosis in India. Fluoride in drinking water samples and urine samples of school children was assessed by a fluoride ion meter. Statistical analysis was done using IBM SPSS Statistics for Windows, Version 25, (Released 2017; IBM Corp., Armonk, New York, United States). Descriptive statistics were used to assess the percentage distribution and Chi-square test of Independence for comparison between variables.

Results

The mean fluoride levels in water in the study areas ranged between 1.5mg/l to 4.2mg/l and there was a prevalence of 44.05% dental fluorosis and 0% skeletal fluorosis in the school children. A total of 48.47% of girls and 40.64% of boys were affected with dental fluorosis. A prevalence of 23.06% mild, 22.1% moderate, 5.31% very mild, and 4.55% severe dental fluorosis was observed in the examined children.

Conclusion

Our study results showed the presence of dental fluorosis with no clinical evidence of skeletal fluorosis among school children in the YSR Kadapa district of Andhra Pradesh. This indicates the fluoride toxicity on teeth in children confirming the exposure to fluoride in the YSR Kadapa district. So, further long-term follow-up surveys are necessary to evaluate skeletal fluorosis in these children though there was no clinical skeletal fluorosis. This highlights that measures need to be enforced by the local governance for the supply of defluorinated water in these areas.

## Introduction

Fluoride intake has both useful and harmful effects on human health and they depend on the quantity and duration of daily uptake. Small doses of around 0.8-1.0 mg/l taken daily through water and food, especially in children below eight years of age help in the normal ossification of tooth enamel and bones [1,2]. As per WHO guidelines [2] and the Bureau of Indian Standards 2012, the permissible limit for fluoride in drinking water is 1mg/l (1PPM) and can be allowed up to a limit of 1.5 mg/l, if there is no alternative water source in the area [3]. Exposure to fluoride doses above 1.5 mg/l per day in children during the teeth formation age may result in teeth fluorosis and beyond 3.0 mg/l per day for about eight to 10 years in both children and adults result in fluorosis of the skeletal system [1,2]. Symptoms of fluorosis vary in different individuals and individual liability to fluorosis varies in different populations with the doses and duration of fluoride exposure [1,2].

The Central Ground Water Board (CGWB) of India after groundwater analysis in various states of India identified and reported areas with fluoride above 1.5 mg/l in groundwater in the districts of Visakhapatnam, West-Godavari, Krishna, Guntur, Prakasam, Nellore, Chittoor, Kadapa, Kurnool, Ananthapur, Srikakulam, and Vizianagaram in the state of Andhra Pradesh [4]. Fourteen fluoride-affected mandalas in a total of 51 mandalas before the bifurcation of the YSR Kadapa district in 2021 were reported to have fluoride levels around 0.34-3.600 mg/l by the CGWB of India on groundwater exploration at the locations from a depth ranging from 107.20 m to 200m below ground level in YSR Kadapa district. These are Simhadripuram, Thondur, Pulivendula, Vemula, Vempalli, Pendlimarri, Veerapunayanupalle, Kamalapuram, Chakrayapet, Lakkireddipalle, Rayachoty, Badvel, Brahmamgarimatem, and Mydukur [4]. The main source of drinking water in these areas is groundwater either in the form of bores or water supply through taps. Geologically, limestone is the most predominant rock of this area in the earth crest, and these rocks have fluoride-bearing minerals that leach out to the groundwater and increase the fluoride concentration in the groundwater [5,6].

The central government of India has identified endemic fluoride areas with high fluoride levels in the YSR Kadapa district and recommended that groundwater in these areas is not suitable for consumption [5]. This prompted us to take up the study to evaluate the toxicity of fluoride in drinking water on the teeth (dental fluorosis) and on the bones (skeletal fluorosis) in school children in endemic fluoride areas in the YSR district as early fluorosis can be easily observed in children’s teeth and bones. The rationale of this study is to bring the present scenario of fluoride levels and its effect on the various organs like teeth and bones in school children in the YSR Kadapa district to the notice of policymakers in the government so that necessary steps are taken to control fluoride levels for the prevention of fluoride related health problems. The study aimed to assess the present scenario on the prevalence of teeth fluorosis and skeletal fluorosis in school children in the YSR Kadapa district. We hypothesize that the fluoride in consumable water in endemic fluoride areas has no toxic effect on teeth and bones in school children in the YSR Kadapa district.

## Materials and methods

A cross-sectional study was carried out among school children in six villages of the YSR Kadapa district to assess the prevalence of teeth fluorosis and skeletal fluorosis in the age group of eight to 14 years. Fourteen mandalas of the YSR district were found with fluoride levels ranging from 0.02 to 4.22 mg/l in groundwater as per the report of the CGWB of India [4] and through various studies conducted by the Geology Department of Yogivemana University, YSR Kadapa district [7,8,9-11]. Six villages from different mandalas in YSR Kadapa district, namely, Veerapalli, Guntapalli, Guntlammayapalli, Sibyala, Rayachoty rural, and Indukurupalle were randomly chosen as study areas. The study areas are tropically dry regions that are wholly agricultural and farming is the main occupation.

Ethical clearance was taken from the Institutional Ethical Committee before the start of the study. Consent was obtained from the District Educational officer, school authorities, and parents of the school children for conducting a school survey for fluorosis among school children. Doctors and healthcare workers in primary health centers near the study areas participated in the survey with the permission of the District Medical and Health Officer, Kadapa. 

This study included children between eight and 14 years of age residing in that area since birth with teeth fully erupted and/or partially erupted (but the crown was more than half) and present on the examination day. The study excluded children with previous dental treatment, suffering from any systemic disease using drugs for a long period, and with no consent from parents or legal guardians. The school surveys were carried out for the estimation of teeth fluorosis and skeletal fluorosis in school children in these rural areas.

**Figure 1:**
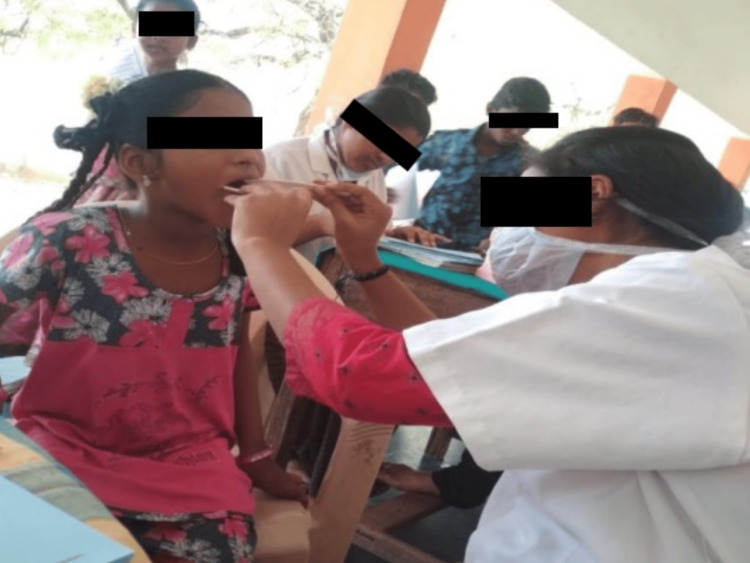
School survey screening for dental fluorosis

The study population comprised 488 schoolchildren. The personal details like age, sex, height, waistline, weight, residence, and socioeconomic status of the subjects were noted through a questionnaire. Further information from participants about education, drinking water sources, water consumption, toothpaste type, number of brushing times per day, and the type of brushing were also collected through interviews. A general physical examination was done by trained doctors, house surgeons, and students. The BMI of the subjects was calculated by height and weight. The subjects were seated in a chair in bright daylight and clinical examination was done in the school compound itself (Figure 1).

Dental fluorosis and skeletal fluorosis were examined as per the norms laid down by the Central Government of India in the National Prevention Program for Fluorosis in India [12]. The presence and severity of fluorosis were assessed as per the Dean’s index and depended on the most severe form of fluorosis found on two or more teeth.

The criteria for assessing skeletal fluorosis as per the National Fluorosis Prevention Programme by the Central Government of India [12] were individuals residing in an area with fluoride above 1.0 mg/l in water with one or more of the health complaints such as pain and stiffness in the neck, shoulder, backbone (lumbar region), knee, and hip, pain in either one or two or more joints, and limited mobility of the cervical and/or lumbar spine that makes the individual turn the whole body toward that side to see, and knock knee/bow leg in children and adolescents. The inability to swim is noticed in the late stage of skeletal fluorosis. Ugly gait and posture are seen in severe skeletal fluorosis.

All the schools surveyed had both public water supply through taps and bore water for drinking purposes. Water samples from all the drinking water sources were collected in 500ml sterile, clean, high-density polyethylene bottles, labeled, coded, and sent to the laboratory for fluoride estimation on the same day. Urine samples were collected in a polyethylene tube (50 mL) from the children and two to four drops of toluene were added as a preservative. Then the samples were transported to the laboratory and stored at 4°C till analysis. 

Water and Urine samples were tested for fluoride ion concentrations using the national standard ion selective electrode method. All reference solutions for the fluoride determinations were deionized water, and all chemicals used in the tests were reagents of analytical purity. Parallel samples were set for measurement and three averages were taken. Fluoride analysis was done by using an electrode ion.

Statistical analysis was done using IBM SPSS Statistics for Windows, Version 25, (Released 2017; IBM Corp., Armonk, New York, United States). Descriptive statistics like percentages, mean, and standard deviation were used to describe the water and urine fluoride levels, the prevalence of teeth fluorosis and skeletal fluorosis, and the grading of dental fluorosis. The chi-square test of independence was done to assess the significance levels in the sex-wise distribution of dental fluorosis. A p-value of <0.05 was considered statistically significant. Pearson correlation coefficient analysis was done to assess the linear relationship between fluoride levels in drinking water and the prevalence of dental fluorosis in various villages and a p-value of <0.05 was considered statistically significant.

## Results

Fluoride levels in drinking water in 6 randomly selected villages in the YSR district are tabulated in Table 1. 

**Table 1:** Fluoride levels in water samples collected from different drinking water sources in six villages in YSR Kadapa District, Andhra Pradesh, India Suffixs 1 to 12 numbers are given for the identification of the samples.

SNo	Village/town	Source of water sample	Water sample	Fluoride level in ppm
1	Veerapalli	Borewell	Water sample 1	3.5
		Hand pump	Water sample 2	3.2
2	Guntapalli	Rural water supply	Water sample 3	1.8
		Borewell	Water sample 4	1.6
3	Guntlammayapalli	Hand pump	Water sample 5	4.2
		Borewell	Water sample 6	4.0
4	Sibyala	Hand pump	Water sample 7	2.7
		Rural water supply	Water sample 8	2.4
5	Rayachoty rural	Municipal water supply	Water sample 9	1.4
		Municipal water supply	Water sample 10	1.7
6	Indukurupalle	Rural water supply	Water sample 11	1.9
		Borewell	Water sample 12	2.5

All six villages were found to have high fluoride levels in drinking water ranging between 1.4 mg/l and 4.2 mg/l, more than the permissible limit by the WHO and the Bureau of Indian Standards, and were selected as study areas.

The prevalence of dental fluorosis and skeletal fluorosis in school children in the YSR district is tabulated in Table 2. 

**Table 2:** Distribution of the study population according to the prevalence of dental fluorosis and skeletal fluorosis among school children in YSR Kadapa district, Andhra Pradesh, India

Children	Dental fluorosis N (n%)	Skeletal fluorosis N (n%)
Present	215 (44.05 %)	0 (0%)
Absent	273 (55.94%)	488( 100%)
Total	488 (100%)	488 (100%)

**Figure 2:**
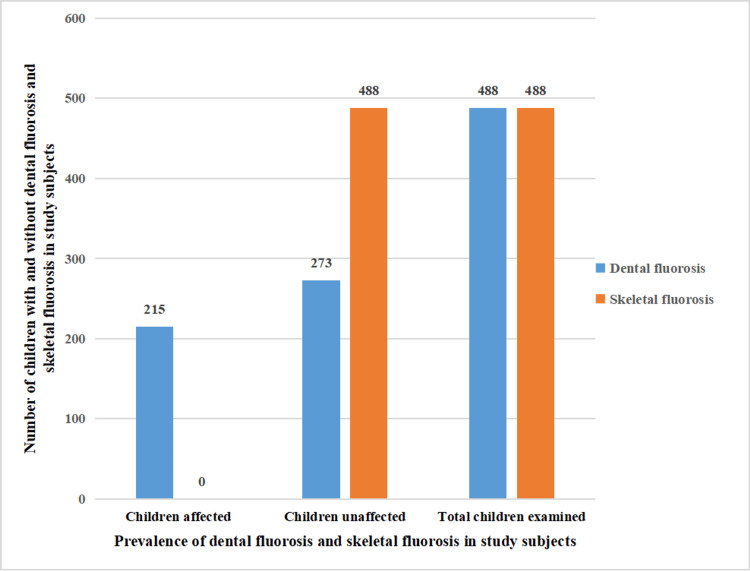
Prevalence of dental fluorosis and skeletal fluorosis in study subjects

The total study subjects were 488 and out of them, 215 (44.05%) were found to have dental fluorosis, and 273 (55.94%) were free from dental fluorosis. There was no clinical case of skeletal fluorosis in school children (Figure 2).

Dental fluorosis in different genders of school children in YSR Kadapa is tabulated in Table 3.

**Table 3:** Distribution of the study population according to the sex-wise prevalence of dental fluorosis in the YSR Kadapa district, Andhra Pradesh, India Chi-square test of independence

Sex	Number of children with dental fluorosis (n%)	Number of children without dental fluorosis (n%)	Total number (n%)	P-value
Boys	102 (48.57%)	108 (51.42%)	210 (43.03%)	p = 0.081061
Girls	113 (40.64%)	165 (59.35%)	278 (56.97%)	
Total	215 (44.06%)	273 (55.94%)	488 (100%)	

Among 488 subjects, 210 were boys (43.03%) and 276 were girls (56.97%). A total of 102 out of 210 boys (48.47%) were found to have dental fluorosis and 113 out of 278 girls (40.64%) were found to have dental fluorosis. 

The comparison of dental fluorosis and fluoride levels in drinking water and urine samples in various villages is tabulated in Table 4. 

**Table 4:** Comparison of prevalence of teeth fluorosis in various villages in the YSR Kadapa district, Andhra Pradesh, India, with varying levels of fluoride concentration in drinking water and urine samples Pearson correlation coefficient (r) test, r = +0.90018 (positive correlation)

S No.	Name of the village	Mean fluoride level in water (mg/l)	Mean fluoride level in urine (mg/l)	Number of children with dental fluorosis (n%)	Total number of children examined
1	Veerapalli	3.35	1.95	38 (67.9%)	56
2	Guntapalli	1.7	0.765	23 (26.1%)	88
3	Guntlammayapalli	4.1	2.13	68 (69.4%)	98
4	Sibyala	2.55	1.75	34 (43.6%)	78
5	Rayachoty Rural	1.5	0.45	15 (19.7%)	76
6	Indukurupalle	2.2	1.12	37 (40.2%)	92
Total				215 (44.05%)	488

A total of 69.4% of dental fluorosis was noticed in Guntlammayapalli (mean 4.1 ppm), and the lowest prevalence of 19.7% was in Rayachoty rural (1.5mg/l) villages. The presence of fluoride in urine samples collected from subjects was noticed in all these villages.

One one-way analysis of variance (ANOVA) test was performed and the p-value for mean fluoride levels in water samples among all the villages was found to be 0.02370 and that of the urine sample was found to be 0.0162.

**Figure 3:**
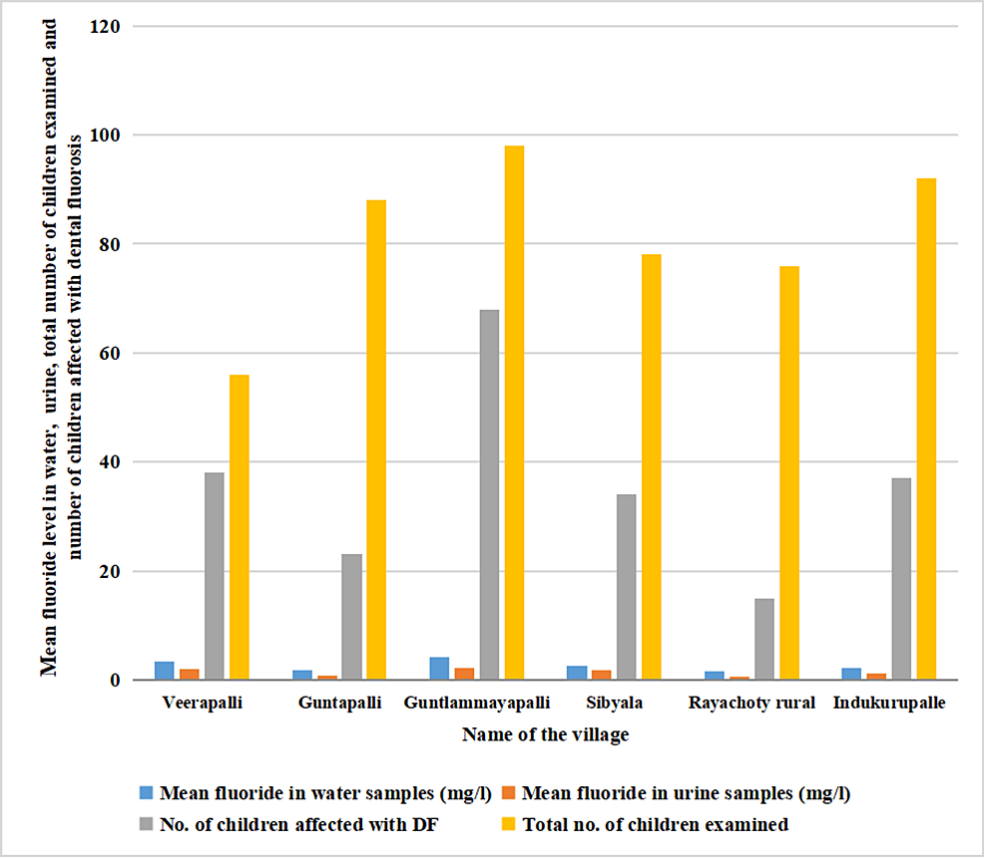
Prevalence of dental fluorosis in the study subjects with fluoride levels in water and urine samples in the YSR Kadapa district, Andhra Pradesh, India DF: dental fluorosis

Pearson correlation coefficient (r) test gave r=+0.90018 (positive correlation) with a p-value of 0.01447. A statistically significant positive correlation was noticed in the occurrence of teeth fluorosis with an increase in fluoride levels in drinking water (Figure 3).

Different grades of dental fluorosis observed in school children as per the Dean’s fluorosis index are tabulated in Table 5.

**Table 5:** Distribution of the study population as per prevalence of different grades of teeth fluorosis in school children in the YSR Kadapa district, Andhra Pradesh, India

S. No.	Dean's index	
	Severity of dental fluorosis	Number (n%)
			1.	Normal	273 (55.94%)
2.	Questionable	-
3.	Very mild	25 (5.31%)
4.	Mild	92( 23.06%)
5.	Moderate	85 (22.1%)
6.	Severe	13 (4.55%)
7.	Total	488 (100%)

**Figure 4:**
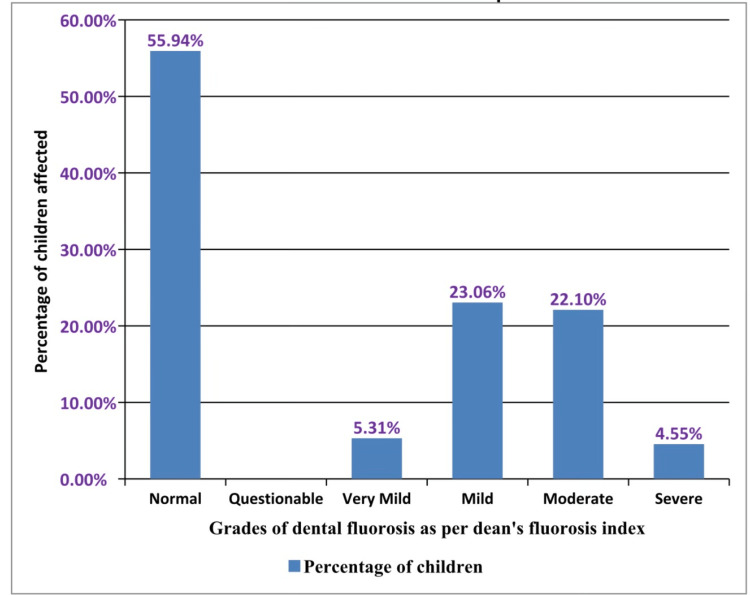
Prevalence of different grades of dental fluorosis as per Dean’s index in school children in the YSR Kadapa district, Andhra Pradesh, India

The majority were seen with mild (23.0%) and moderate (22.1%) forms. The very mild form was found to be 5.31% and the severe form was about 4.55%. The questionable type was not noticed (Figure 4).

**Figure 5:**
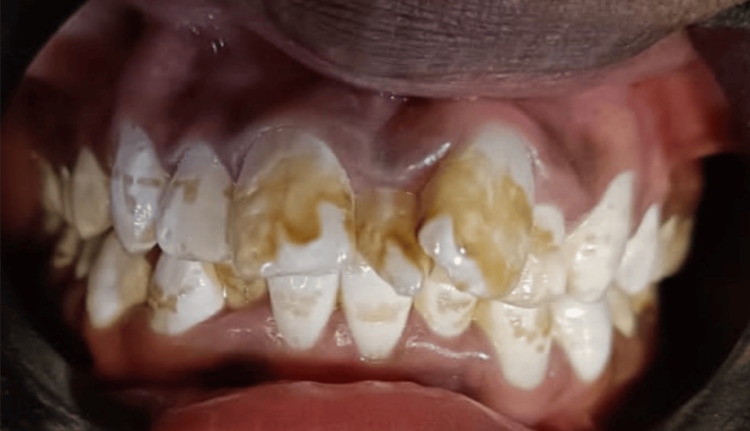
Clinical photograph showing severe dental fluorosis

**Figure 6:**
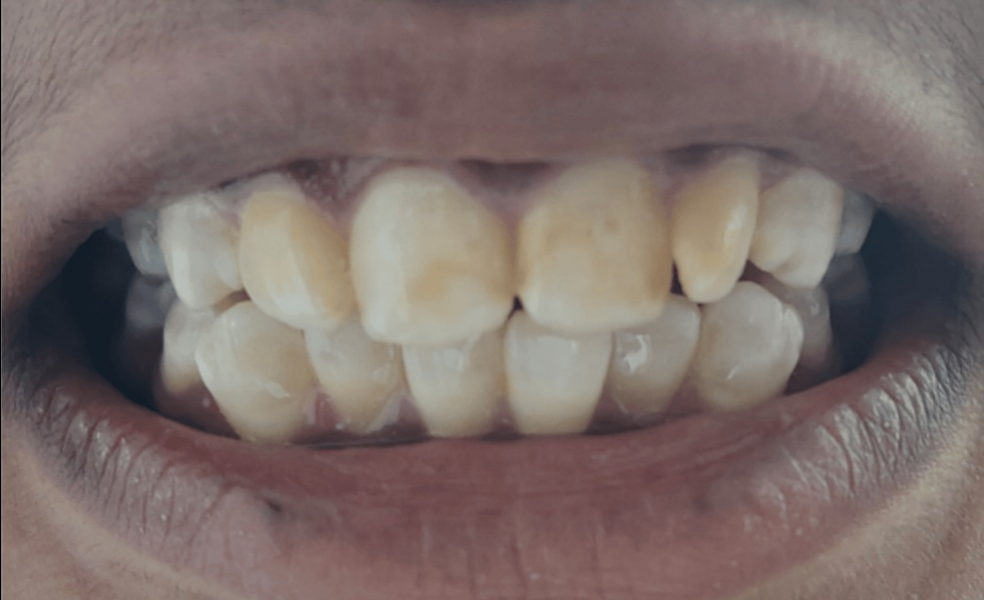
Clinical photograph showing moderate dental fluorosis

**Figure 7:**
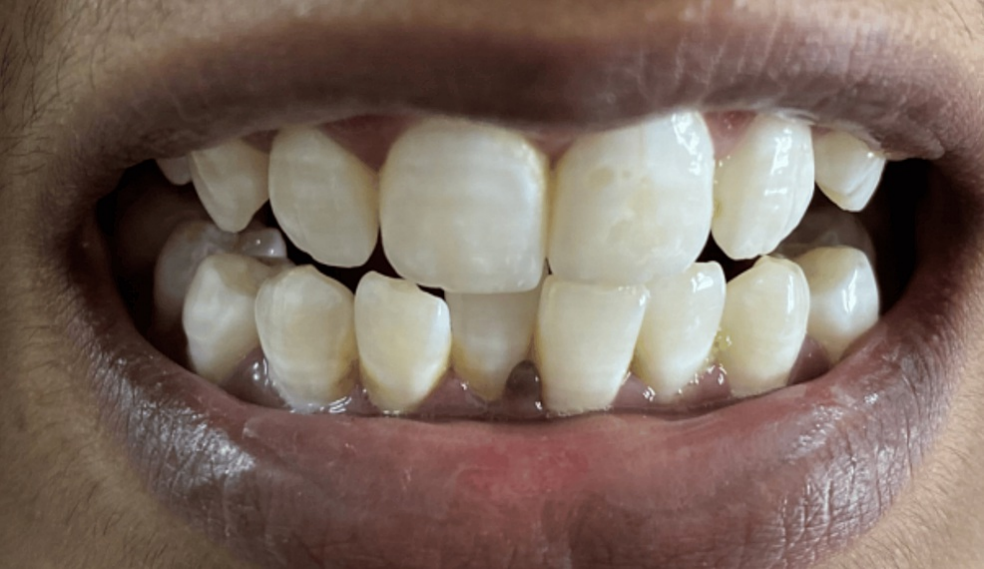
Clinical photograph showing mild dental fluorosis

**Figure 8:**
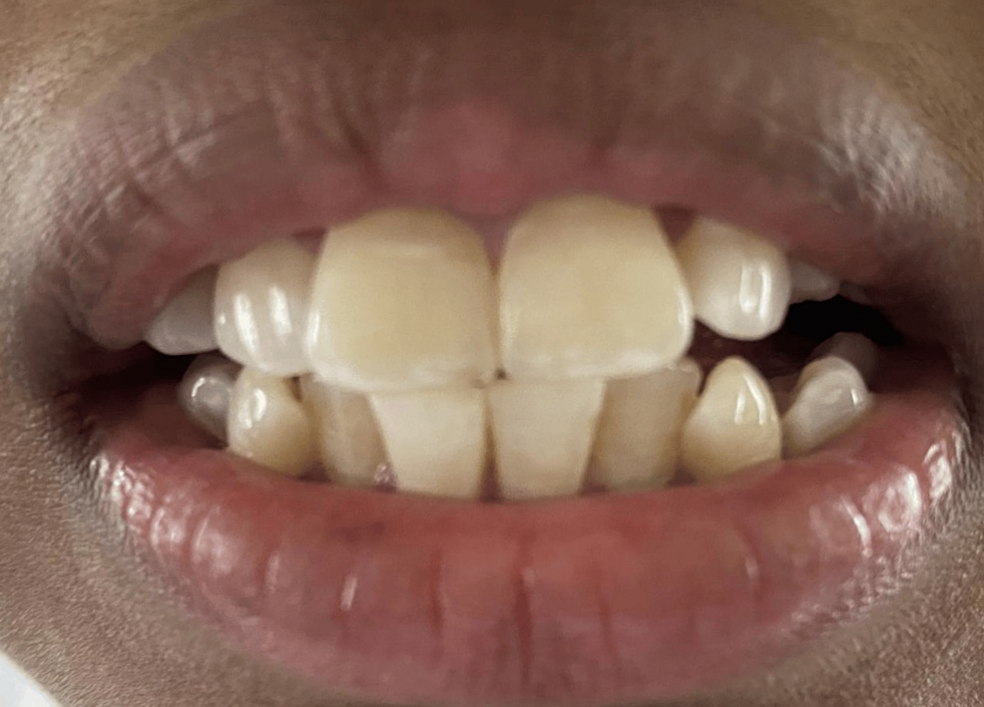
Clinical photograph showing very mild dental fluorosis

Different grades of dental fluorosis are shown in the following clinical photographs: Figure 5 - severe dental fluorosis, Figure 6 - moderate dental fluorosis, Figure 7 - mild dental fluorosis, and Figure 8 - very mild dental fluorosis.

## Discussion

Six villages from 14 mandalas were randomly chosen and selected as study areas in this study. The mean fluoride levels in the water samples (WS1-12) collected from the study areas were found to be between 1.5 to 4.1 mg/l. The results of our study were consistent with the report by the CGWB of India (2015) in which the groundwater fluoride level in the YSR Kadapa district ranged from 2.890-3.60 mg/l and concluded that this groundwater consumption was harmful to humans. No man-made pollution was observed as there are no fluoride pollutant-related industries in the district and so fluoride contamination in the groundwater is a natural process in the YSR district. The probable factors for the fluoride in underground water in study areas in YSR Kadapa district could be due to the semi-arid climate with high temperatures, low average annual rainfall of about 700mm, leaching of fluorine-bearing minerals from granite rocks, and generally alkaline nature of the soil, and the fact that the main rivers Sagileru and Penna in the district are not perennial rivers [5]. Rayachoty is one among the 23 acutely drought-hit locations in the barren Rayalaseema district, where the groundwater levels are very deep, about 2,000 feet, and they also have more fluoride levels [5,6]. 

In the present study, mean fluoride ion levels in the water samples tested in the randomly chosen six different villages ranged from 1.5-4.1 mg/l. A total of 44.05% of children examined were found to have dental fluorosis in the study areas. The findings in this study are in correlation with the studies conducted by Sebastian et al. in Mysore, India [13] and by Khan et al. in Pakistan [14], which concluded that the risk of fluoride accumulation increases in hot tropical parts of the world due to the consumption of more water by people [13,14]. Consumption of drinking water by the human body usually depends on the atmospheric temperature. Thus, extremely high temperatures ranging from 38°C to 48°C in the study area during the summer season necessitate increased consumption of water per day. This leads to more uptake of fluoride which in turn results in more accumulation of fluoride in the human body and more prevalence of dental fluorosis. This could be the reason for the occurrence of dental fluorosis in children even at 1.5 mg/l as these areas have moderately high temperatures throughout the year [5,6] necessitating more consumption of water. 

In this study, there was no clinical evidence of skeletal fluorosis on general physical examination in school children in the study areas. Our study results of (0%) prevalence of skeletal fluorosis in the selected areas with mean fluoride levels ranging between 1.5mg/l to 4.1mg/l are consistent with the results of Choubasia [15] who noted the absence of skeletal fluorosis in children encountering fluoride levels at 3.2ppm, 3.7ppm, and 4.00ppm. Our study results also are consistent with Jin Cao et al.'s [16] study results, even though they observed none of the children showed any overt clinical features of skeletal fluorosis by physical examination; on the examination of the radiographs, developmental skeletal abnormalities were noticed, which might represent an early stage of skeletal fluorosis. The reason for the 0% prevalence of clinically evident skeletal fluorosis could be either that skeletal fluorosis was a sub-clinical presentation or that there was no effect of fluoride on the skeletal system in these children as the fluoride may get deposited as people get older. Either of the reasons could have been confirmed by skeletal radiographs; it was not possible to take radiographs of the children in the present study due to noncooperation from the parents of the children.

Mixed results were observed in different studies on the gender-wise occurrence of dental fluorosis in children as discussed by Akuno et al. [17]. Studies by Keçeci et al. [18], Saldarriaga et al. [19], and Verma et al. [20] showed no relationship between gender and fluoride effect on teeth, while some other studies showed significant gender differences in the prevalence of teeth fluorosis. In the present study, more prevalence was observed in girls than in boys. Though the differences were not statistically significant, the study result was consistent with the observations of Gopalakrishnan et al. [21], Arvind et al. [22], and Chauhan et al. [23], where female predominance was noted. The reason girls suffered more from dental fluorosis than boys was not clear and this might be due to poor nutrition among girls. No studies have explained the difference in association between dietary patterns and fluorosis. Liu et al. [24], through their research, stated that diet might influence the occurrence of fluorosis in men and women based on their sex hormones, or sex-specific genes, which are related to the control of fluorosis. The study observation was contrary to the results of the studies by Sudhir et al. [25], Visalrao et al. [26], etc., where the observed prevalence was higher in boys than girls. This male predominance was explained in a study by Houari et al. [27] as the high testosterone levels in males following birth, concomitant with amelogenesis, may cause a sexual dimorphism in enamel quality. Future studies are needed to further investigate this observation of sexual dimorphism.

Further, it was noticed that the occurrence of dental fluorosis ranges from 19.7% to 69.4%. The highest occurrence of dental fluorosis was seen in the Guntlammayapalli village where the mean fluoride level was 4.1mg/l and the lowest prevalence is in Rayachoty rural where the mean fluoride level was 1.5mg/l. Statistically, a significant positive correlation (r=+0.90018) was noticed between the prevalence of dental fluorosis and fluoride levels in drinking water, that is, the occurrence of dental fluorosis increases with the increase in the fluoride in drinking water. In this study, it was observed that the percentage of children affected with dental fluorosis increased with an increase in fluoride level in drinking water, which is consistent with the results of the studies done by Viswanathan et al. [28], Mandinic et al. [29] and Rango et al. [30], wherein it was concluded that teeth fluorosis increases with the concentration of fluoride in drinking water. Fluoride in urine samples collected from the children confirmed that all the children were exposed to fluoride.

In this study, more prevalence of mild dental fluorosis and moderate dental fluorosis compared to very mild dental fluorosis and severe dental fluorosis was observed in children between eight to 14 years of age. These results are consistent with the observations of Sarvaiya et al. [31] and Rwenyonyi et al. [32], that in children between eight to 12 years of age, the prevalence of predominant grades of dental fluorosis was mild and moderate. Surprisingly, no clinical single case of the questionable type of fluorosis was noticed in our study. The severity of dental fluorosis increased with the age of the children. Thylstrup et al. (1978) [33] and Baelum et al. (1986) [34] stated that the severity of fluorosis increases with age, an observation which could be explained as a high fluoride concentration environment disturbs the ameloblasts during the development of the enamel in creating regular enamel rods, resulting in subsurface porosities. These subsurface porosities might change into pitting and elevate the definition of fluorosis with time. The information from the questionnaire in the present study also observed that there were differences in certain habits in the children of younger age compared to those of older age such as the force and duration of brushing, type of toothbrushes (with hard bristles), and dietary habits that might result in damage to unsupported hypo calcified enamel (subsurface porosities) in enamel fluorosis teeth, increasing severity of teeth fluorosis with an increase in age.

Thus, our study results showed a significant positive correlation between the fluoride concentration in drinking water and urine and the prevalence of dental fluorosis in the children in the YSR Kadapa district. These children were found to be residing in these places since birth and got exposed to fluoride in drinking water during the time of permanent teeth formation and enamel maturation, reflecting dental fluorosis. These findings are consistent with the findings of Jarquín-Yañez et al. [35] and suggest that fluorosis in the village children is mainly due to taking fluoride through drinking the groundwater.

Limitation

The fluoride levels in the groundwater are related to many factors and vary from time to time and so does the fluoride toxicity. Our study was a cross-sectional investigation done on school children aged between eight to 14 years only at a point in time. The observed fluoride toxicity on teeth and not on bones in the school children was only the indication of endemic fluorosis in the rural YSR district. To verify the appropriate effect of fluoride on teeth and bones and to better establish the fluoride toxicity, the study strongly suggests the continuous extensive screening of children of all age groups for fluorosis and groundwater sources in YSR Kadapa district for fluoride levels, along with the regular monitoring of urine samples for a bigger sample size.

## Conclusions

So, in conclusion, it is clear that the fluoride concentration in consumable water was higher in rural areas of the YSR Kadapa district than the recommended upper limit by the WHO. There are endemic fluoride areas in the YSR Kadapa district. The prevalence of dental fluorosis was noticed among school children and confirms the toxic effect of fluoride on teeth. However, the observation of no clinical skeletal fluorosis in school children needs to be followed up. Thus, it has been determined that the Kadapa district requires a fluoride management plan for the supply of defluorinated drinking water to the studied areas. To certify the exact prevalence rate of dental fluorosis and skeletal fluorosis, more research is required. Continued studies are a must to confirm the results of the present study.
